# Sawdust waste as a low-cost support-substrate for laccases production and adsorbent for azo dyes decolorization

**DOI:** 10.1186/s40201-016-0244-0

**Published:** 2016-01-20

**Authors:** Dalel Daâssi, Hela Zouari-Mechichi, Fakher Frikha, Susana Rodríguez-Couto, Moncef Nasri, Tahar Mechichi

**Affiliations:** Laboratory of Enzyme Engineering and Microbiology, Ecole Nationale d’Ingénieurs de Sfax, University of Sfax, Route de Soukra Km 4.5, BP 1173, Sfax, 3038 Tunisia; Department of Biology, Faculty of Sciences and Arts, Khulais, University of Jeddah, Jeddah, Saudi Arabia; Faculty of Sciences, University of Sfax, Route de Soukra Km 4,5, Sfax, 3000 Tunisia; CEIT, Unit of Environmental Engineering, Paseo Manuel de Lardizábal 15, 20018 San Sebastian, Spain; IKERBASQUE, Basque Foundation for Science, Alameda de Urquijo 36, 48011 Bilbao, Spain

**Keywords:** SSF, Sawdust, *C. gallica*, Optimization, Decolorization

## Abstract

**Background:**

Laccases are multicopper oxidases with high potential for environmental and industrial applications. Low-cost laccase production could be achieved by solid state fermentation on agro-industrial by-products.

**Methods:**

A number of agro-industrial solid wastes were tested as support-substrate for laccase production by *Coriolopsis gallica* under solid-state fermentation (SSF) conditions. Response surface methodology (RSM) was used to optimize the medium composition for laccase production. Initial screening by Plackett-Burman design was performed to select the major variables out of 20 tow medium components fellowing this Central composite design (CCD) was employed to optimize the level of the selected variables.

**Results:**

Sawdust waste was shown to be the best support-substrate for laccase production by the *C. gallica*. Peptone as source of organic nitrogen, Cd^2+^ as laccase inducer and liquid/solid (L/S) ratio were found to have significant effects on laccase production. Operating at optimum concentrations of the most significant variables (peptone, 4.5 g L^−1^, L/S ratio, 5.0 and Cd^+2^ 1.0 mM) extracellular laccase activity was enhanced from 1480 U L^−1^ (60.5 U g^−1^), to 4880 U L^−1^ (200 U g^−1^) which meant a 3.2-fold increase in laccase activity. On the other hand, sawdust waste was studied as a low cost adsorbent to remove the azo dyes Reactive Black 5 (RB5) and Acid Orange 51 (AO51). Decolorization percentages around 67 and 75 % were obtained in 24 h for RB5 and AO51, respectively.

**Conclusion:**

When used as a support substrate, sawdust yielded the highest laccase production which was increased 3.2 times using RMS optimization.

## Background

Laccases (p-diphenol: dioxygen oxidoreductases; EC 1.10.3.2) are particularly abundant in white-rot fungi, which are the only organisms able to degrade the whole wood components [1]. Most studies on laccase production by white-rot fungi have been performed in liquid cultures, which do not reflect the natural living conditions of such fungi (wood). In solid-state cultures, white *Coriolopsis* is assumed to be one of the most efficient lignin degraders -rot fungi grow under conditions close to their natural habitat. This may allow them producing certain enzymes and metabolites, which usually would not be produced or would only be produced at a low yield in submerged fermentation (SmF) conditions [2]. Agricultural and food wastes could be used as support-substrates to produce bulk chemicals and products with high commercial value like organic acids, proteins, alcohol and enzymes [3] by microorganisms. Furthermore, the utilization of these compounds helps in solving the pollution caused by their disposal [4].

Fungal growth in SSF is different from fungal growth in SmF because of different surface phenomena, moisture content and chemical composition of the support-substrate [5]. SSF provides a better oxygen circulation; it is a static process without mechanical energy consumption and reproduces the natural living conditions of the white-rot fungi [4]. SSF constitutes an interesting alternative for fungal cultivation since the metabolites so produced are more concentrated, purification procedures are less costly, less wastewater is formed, product recovery is easier, bacterial contamination is lessened and is a simple process [6].

Two types of SSF systems can be differentiated depending on the nature of the solid support used. The first one involves cultivation on an inert support (synthetic material) [7] whereas, the second and most commonly-used system involves cultivation on a natural support (organic material) [8]. Many studies reported the exploitation of SSF to give an extra value to agro-industrial residues [9, 10] and different reactors for SSF were described [11].

In SSF, the selection of a suitable solid support-substrate is a critical factor. Ligninolytic enzymes have been successfully produced by SSF using various agro-industrial wastes such as banana waste [12], potato peelings [13], barley bran [14], wheat bran [8] and kiwi fruit [13]. In addition, the composition of the production medium is a key parameter in optimizing SSF processes because nutritional factors, such as the nature and concentration of carbon and nitrogen source and trace metals, can influence the growth and production of metabolites. Researchers are encouraged to apply statistical experimental approaches, e.g. Plackett-Burman (PB) design and response surface methodology (RSM), which provide a great amount of information based on only a small number of experiments [15].

It is very interesting to find new ways of producing laccase with higher activities at lower cost due to the enormous potential that this enzyme offers for the development of efficient biotechnological applications. Such applications include the detoxification of industrial effluents, mostly from textile-dyeing industry, which is considered as the most polluting one amongst all industrial sectors [16]. Effluents discharged from dyeing industries are highly colored and represents a serious concern worldwide since they contain different kinds of synthetic dyes. The use of dyes has generated much concern due to their toxic effects, since they have been reported to cause carcinogenesis, mutagenesis, chromosomal fractures, teratogenecity and respiratory toxicity [17].

Biosorption (adsorption) by lignocellulosic materials may be an alternative method for removing dyes from effluents. Several studies have shown the biosorption potential of different lignocellulosic materials [18, 19]. Because dye-adsorbed lignocellulosics create another source of pollution, adsorption of dyes onto lignocellulosic materials does not completely solve this problem. In light of this situation, a two-step method combining biosorption and SSF may be an alternative approach and effective solution. The combination of these two methods, biosorption and biodecolorization by SSF, has potential to be a suitable process for dye removal and laccase production. Sawdust is an abundant by-product of the wood industry that is either used as cooking fuel or as packing material. Sawdust is easily available in the countryside at zero or negligible cost [20]. It contains various organic compounds (lignin, cellulose and hemicellulose) with polyphenolic groups that might be useful for binding dyes through different mechanisms [21, 22].

The aims of this study were: (a) to assess the potential of selected agro-wastes for laccase production by the white-rot fungus *C. gallica* under SSF conditions, (b) to optimize the laccase production by *C. gallica* under SSF conditions operating with sawdust waste as support-substrate using CCD and (c) to test the ability of the ligninolytic complex secreted in SSF conditions for azo dyes biodecolorization.

## Methods

### Chemicals

All chemicals were of certified reagent analytical grade. The following azo dyes were used: Reactive Black 5 (RB5) and Acid Orange 51 (AO51). They were purchased from Sigma-Aldrich (St. Louis, MO, USA). The characteristics of the dyes are summarized in Table 1. Stock dye solutions (0.1 % *w/v* in water) were stored in the dark at room at room temperature.

**Table 1:** Characteristics of the azo dyes used

Dye	CI^a^ number	λ_max_ (nm)	Chemical structure
Reactive Black 5 (RB5)	20505	597	
Acid Orange 51 (AO51)	26550	450	

^a^ *CI* color index

### Support-substrates

Tip of palm (3 × 3 × 2 mm^3^), oatmeal, wheat bran, straw (3 × 3 × 2 mm^3^), filer paper (size 2 × 2 × 1 mm^3^), sawdust (3 × 2 × 2 mm^3^), orange peelings (size 5 × 5 × 2 mm^3^), melon peelings (size 5 × 5 × 2 mm^3^), wood (2 × 3 × 2 mm^3^) and olive leaves (4 × 2 × 2 mm^3^) were used in this study as support-substrate under SSF conditions for laccase production by *C. gallica.*

Prior to use, the peelings of orange and melon were pre-treated as follows: they were first soaked for 1 h in 30 mL of 0.1 M KOH (10 g of fresh peelings) to neutralize organic acids [23]. Then, they were thoroughly washed with distilled water and dried at 30 °C for 2 days. The peelings were autoclaved at 120 °C for 1 h. The other support-substrates were used in dry state.

### Fungal strain, media and culture conditions

The fungal isolate used in this study was *C. gallica*. The isolate is maintained in the culture collection of our laboratory in the Ecole Nationale d’Ingénieurs de Sfax (Tunisia). For short-term conservation, the isolate was cultured on 2 % malt extract and 1.5 % agar in Petri dishes at 30 °C for 4 days and stored at 4 °C until used.

*C. gallica.* was cultured in 250-mL Erlenmeyer flasks containing 5.0 g of the substrates. These substrates were hydrated with 15 mL of 50 mM acetate buffer (pH 5.0). Inoculation was carried out directly in the Erlenmeyer flasks. Three plugs (diameter, 3 mm), from a 5-day growing fungus on malt extract agar (MEA) per Erlenmeyer were used as inoculum. The cultures were supplemented with laccase-inducing compounds (solution sterilized separately 60 mM) at the beginning of the cultivation. The Erlenmeyer flasks were incubated statically for 10 days in complete darkness at 30 °C.

### Laccase activity determination

Laccase activity was measured by monitoring the increase in absorbance at 469 nm (ε_469nm_ = 27,500 M^−1^ cm^−1^) of a reaction mixture containing 10 mM 2,6-dimethoxyphenol in 100 mM acetate buffer, pH 5.0 [24]. Enzymatic reactions were carried out at room temperature (22–25 °C). One unit of enzyme activity was defined as the amount of enzyme oxidizing 1 μmol of substrate per min. The activities were expressed in U L^−1^.

### Plackett-Burmann design

Several parameters in the culture medium can affect laccase production. In order to identify these parameters, different components of the culture medium were evaluated using the PB experimental design [25]. The total number of trials to be carried out according to Plackett-Burmann is k + 1, where k is the number of variables. Each variable is represented at two levels, high and low which are denoted by “+” and “−”, respectively; and also by five central points. The effect of each variable on each response was determined by subtracting the average response of the low level from that of the high level. Table 2 illustrates the factors under investigation as well as the levels of each factor used in the experimental design. Design expert, version 7.0 (STAT-EASE Inc., Minneapolis, USA) was used to analyze the experimental PB design. In our experiments, the variables with confidence levels above 95 % were considered as influencing laccase production significantly.

**Table 2:** Range of different variables studied in the Plackett-Burmann design

Factors				levels		
Variables		Units	Variable code	Low	Central point	High
				(−)	(0)	(+)
Carbon sources (F1)	Glucose	g L^−1^	X1	0	10	20
	Maltose	g L^−1^	X2	0	10	20
	Saccharose	g L^−1^	X3	0	10	20
	Starch	g L^−1^	X4	0	10	20
Nitrogen sources (F2)	Casein Peptone	g L^−1^	X5	0	10	20
	NH_4_SO_4_	g L^−1^	X6	0	2.5	5
	KNO_3_	g L^−1^	X7	0	2.5	5
	Urea	g L^−1^	X8	0	2.5	5
Phosphate (F3)	KH_2_PO_4_	g L^−1^	X9	0	0.025	0.05
MgSO4 (F4)	MgSO_4_	g L^−1^	X10	0	0.25	0.5
Inductors (F5)	CuSO_4_	mM	X11	0	0.5	1
	Ethanol	mL L^−1^	X12	0	15	30
	Cd^2+^	mM	X13	0	0.5	1
	MnSO_4_	mM	X14	0	0.5	1
	Ferulic Acid	mM	X15	0	0.5	1
	Vanilllic acid	mM	X16	0	0.5	1
Buffer (F6)	Acetate-Na	mM	X17	0	10	20
	Citrate-Na	mM	X18	0	10	20
	Tartrate-Na	mM	X19	0	10	20
pH (F7)	pH		X20	3	4	5
Inoculum nombre (F8)	Inoculum	5 mm	X21	4	6	8
Liquid/solid ratio (F9)	L/S ratio		X22	1.5	4.75	8

### Response surface methodology

RSM was used to optimize the screened components that enhanced laccase production using the CCD [26]. Basically this optimization process involves three major steps: performing the statistically designed experiments, estimating the coefficients in a mathematical model and predicting the response and checking the adequacy of the model. Using the mathematical model, the levels of the variables giving maximum response can then be calculated. Each factor in the design was studied at five different levels (−α, −1, 0, +1, +α).

A set of 20 experiments was performed. All the variables were taken at a central-coded value considered as zero. The minimum and maximum ranges of variables investigated and the full experimental plan with respect to their values in actual and coded form is listed in Table 3. Upon completion of experiment, the laccase production was taken as dependent variable or response (Y). The independent variables are coded for statistical calculation according to the following Eq. (1):

1 $$ Xi=\left( xi-x0\right)/ xi $$

**Table 3:** The range and the levels of the variables

Factor	Variable	Unit	Range and level of actual and coded values				
			−α	−1	0	1	α
peptone	A	g L^−1^	0.688	2	4.5	7	8.311
Cd	B	mM	0.237	0.5	1	1.5	1.762
L/S	C	–	3.475	4	5	6	6.524

Where Xi is the dimension less coded value of the independent variable xi, xi is the real value of that independent variable, x0 is the real value of that independent variable xi at the center point, △xi is the step change. The role of each variable, their interactions, and statistical analysis to obtain predicted yields is explained by applying the following quadratic Eq. (2):

2 $$ \begin{array}{cc}\hfill \mathrm{Y}={\upbeta}_0+\sum {\upbeta}_i{\mathrm{X}}_i+\sum {\upbeta}_{ij}{\mathrm{X}}_i{\mathrm{X}}_j+\sum {\upbeta}_{ii}\hfill & \hfill {X}_i^2\hfill \end{array} $$

Where Y is predicted response, Xi and Xj are the levels of the independent variables.

Statistical analysis of the model was performed to evaluate the analysis of variance (ANOVA). Statistical significance of the model equation was determined by Fisher’s test value, and the proportion of variance explained by the model was given by the multiple coefficient of determination, R squared (R^2^) value. For each variable, the quadratic models were represented as contour plots (3D) and response surface curves were generated. Design expert, version 7.0 (STAT-EASE Inc., Minneapolis, USA) was used in this investigation.

### Decolorization batch experiments of dyes-adsorbed sawdust

Batch decolorization experiments of azo dyes were performed after 10 days of fungal cultivation, as described in the SSF, sampling and extraction studies sub-section. Each decolorization cycle consisted of the addition of 2 mL of the azo dye (50 mg L^−1^, final concentration) to the cultures. A new cycle was initiated after 24 h when no further change in the dye decolorization was detected. The experiments were performed in three-replicates and reported values are representative of three experiments.

A biotic control (without dye) and an abiotic control (without fungus) were conducted in parallel.

Samples were taken at the beginning of the process and at determined intervals, centrifuged (10,000 × g, 5 min) using a microcentrifuge (MSE Micro Centaur) and the residual dye concentration was spectrophotometrically measured from 500 to 700 nm and calculated by measuring the area under the plot. This approach takes into account the conversion of the dye molecules to other compounds absorbing at different wavelengths and then, the ratio of the area under the visible spectrum is always equal or lower than the ratio of the absorbances at the peak. The absorbances were corrected by subtracting the absorbance of culture without dye. The physical adsorption of the dye to the support was evaluated. Dye decolorization was expressed in terms of percentage according to sani et al. [27]. Biological decolorization was determined by subtracting the decolorization due to the physical adsorption of the dye to the support from the total decolorization.

## Results and discussion

### Selection of the support-substrate

Firstly, a series of experiments were carried out to select the best support-substrate for laccase production by *C. gallica* under SSF conditions. All the tested support-substrates facilitated the attachment of the fungus to them whereas their content in lignin, cellulose, hemicelluloses and sugars provided the required nutrients for fungal growth.

**Fig. 1:**
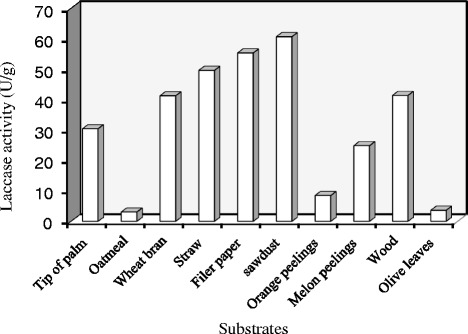
Effect of different support-substrates on laccase production by *C. gallica* under solid-state fermentation (SSF) conditions (10 days)

The profile of laccase production obtained on a particular cultivation day (10 days) using various support-substrates by *C. gallica* under SSF conditions is given in Fig. 1. The first sign of growth was seen on the 2nd or 3rd cultivation day. Laccase activity depended on the type of industrial solid waste used. There was a rapid increase in laccase activity in both sawdust and filter paper cultures during the first 10 days of fermentation with maximum laccase activities of 1480 U L^−1^ for the former and 1300 U L^−1^ for the latter on day 12.

### Plackett-Burmann design

PB design was used to identify which variables had significant effects on laccase production by *C. gallica.* under SSF conditions using sawdust as support-substrate.

As shown in Table 2, 9 factors including 22 medium components were screened in PB experiments. The medium components screened consisted of different carbon and nitrogen sources, KH_2_PO_4_, MgSO_4_, inducers of laccase activity, buffers, pH, inoculum size and liquid/solid (L/S) ratio. The upper and the lower limits of each variable were chosen to encompass the range in literature and to reflect what was done in practice after a preliminary investigation of the limits.

**Fig. 2:**
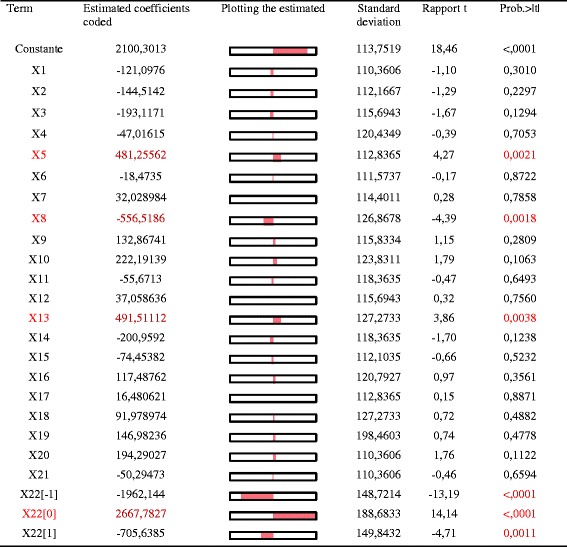
Effects of variables in Plackett-Burman design on laccase production by *C. gallica* under solid-state fermentation (SSF) conditions operating with sawdust as support-substrate

Figure 2 shows the effects of the variables on the response and significant levels. Confidence levels were accepted only when these were above 95 % (*p* < 0.05). Based on the statistical analysis, the factors having the greatest impacts on the production of laccase by *C. gallica*. under SSF conditions using sawdust as a support-substrate were identified as X5 (casein peptone), X8 (urea), X13 (Cd^2+^) and X22 (L/S ratio). These components could be ranked as ratio L/S > Cd^2+^ > casein peptone > urea. The negative influence of urea was significant set at their high levels. Effects of the X5, X13 and X22 were all positive since laccase production increased with increasing concentrations.

In SSF, the concept of water availability or water solvent is vital because it determines the metabolism of microorganisms on solid matrices [28]. In this study humidity environment (L/S) was the most important factor influencing the production of laccase. Indeed, the production of laccase was much higher for a L/S ratio of 4.75 than for one of 1.5. Similar results were described by Rosales et al. [29].

Cd^2+^ (X13) had an acute effect on laccase activity. Thus, an increase of laccase activity was observed in cultures for samples containing high levels of Cd^+2^ (1 mM). This phenomenon was already reported by Baldrian and Gabriel [30], who showed that the activities of endo-1,4-β-glucanase, 1,4-β-glucosidase and laccase from *Pleurotus ostreatus* highly increased in the presence of Cd^+2^ at 2 mM (18.5-fold increase of laccase activity).

The source of organic nitrogen employed had an important effect on laccase synthesis. Thus, when using casein peptone (X5) maximal laccase activities were obtained. Laccase production was higher in the absence of glucose (X1) than with 20 g L^−1^ of glucose. Such negative effect of glucose on the production of laccase has been already described [31]. Therefore this factor was fixed at its lowest level. MgSO_4_ (X10), KH_2_PO_4_ (X9), pH (X20), inoculum size (X21) and the nature of the buffer (sodium acetate (X17), tartrate (X18) or citrate (X19)) had a positive effect on the production of laccase but was not statistically significant as shown in Fig. 2.

All these remarks are summarized in Table 4. As a result, true values of the variables (excluding the three significant variables giving positive effect) in the medium were determined as follows : glucose as carbon source, 5.0 g L^−1^; MgSO_4_ · 7H2O, 0.25 g L^−1^; KH_2_PO_4_, 0.025 g L^−1^; inoculum number, 6.0 plugs; sodium acetate as buffer, 25 mM, pH 5.0. The levels of three significant factors (casein peptone (X5), Cd^2+^ (X13) and L/S ratio (X22)) were determined from the optimization of operating conditions for the production of laccase.

**Table 4:** CCD matrix of three variables with experimental values of laccase production

	Factor 1	Factor 2	Factor 3	Response R1	
Run	A:peptone	B:Cd	C:L/S	Experimental value	Predicted value
1	2.00	0.50	4.00	1778.00	1355.47
2	7.00	0.50	4.00	1490.90	1624.97
3	2.00	1.50	4.00	1036.36	788.91
4	7.00	1.50	4.00	3272.72	3179.23
5	2.00	0.50	6.00	1254.54	969.45
6	7.00	0.50	6.00	1709.09	1577.96
7	2.00	1.50	6.00	936.36	423.72
8	7.00	1.50	6.00	3109.09	3153.05
9	0.69	1.00	5.00	1036.36	1828.13
10	8.31	1.00	5.00	4254.54	4114.21
11	4.50	0.24	5.00	1045.45	1336.75
12	4.50	1.76	5.00	1745.45	2105.58
13	4.50	1.00	3.48	1990.90	2232.83
14	4.50	1.00	6.52	1509.09	1918.60
15	4.50	1.00	5.00	4327.30	4396.37
16	4.50	1.00	5.00	4600.00	4396.37
17	4.50	1.00	5.00	4327.30	4396.37
18	4.50	1.00	5.00	4745.45	4396.37
19	4.50	1.00	5.00	4763.64	4396.37
20	4.50	1.00	5.00	4054.54	4396.37

### Response surface methodology

RSM was used to optimize the nutrient medium for the production of laccase by *C. gallica* using the CCD. The responses of the CCD design were fitted with a second-order polynomial Eq. (2). Except for the linear term A (*p* < 0.05) and the quadratic term A2 (*p* < 0.05), none of the other linear, quadratic and interaction terms were statistically significant (Table 4). The overall second-order polynomial equations for laccase production in expressed in coded variables (3) and actual variables (4) are:

3 $$ \begin{array}{l}\left(\mathrm{Y}\right)=\kern0.5em +4396.37+749.71\ast \mathrm{A}+252.13\ast \mathrm{B}-103.05\ast \kern0.5em \mathrm{C}+530.21\ast \mathrm{A}\ast \mathrm{B}+\\ {}84.75\ast \mathrm{A}\ast \kern0.5em \mathrm{C}+5.21\ast \mathrm{B}\ast \mathrm{C}-613.11\ast \mathrm{A}2-1150.84\ast \mathrm{B}2-998.32\ast \mathrm{C}2\end{array} $$

4 $$ \begin{array}{l}\left(\mathrm{Y}\right)=-25766,5+589,1\ast \mathrm{peptone}+7750,2\ast \mathrm{C}\mathrm{d}+9717,2\ast \frac{\mathrm{L}}{\mathrm{S}}+424,2\ast \\ {}\mathrm{peptone}\ast \mathrm{C}\mathrm{d}+33,9\ast \mathrm{peptone}\ast \frac{\mathrm{L}}{\mathrm{S}}+10,4\ast \mathrm{C}\mathrm{d}\ast \frac{\mathrm{L}}{\mathrm{S}}-98,1\ast {\mathrm{peptone}}^2-4603,4\ast \\ {}{\mathrm{Cd}}^2-998,3\ast \frac{{\mathrm{L}}^2}{{\mathrm{S}}^2}\end{array} $$

Where, Y is laccase activity (U L^−1^); A is casein peptone (g L^−1^); B is Cd^2+^ (g L^−1^). The statistical significance of the model equation was evaluated by the F-test for analysis of variance (ANOVA), which showed that the regression was statistically significant at 99 % (*p* < 0.05) confidence level. The model F-value of 20.5 for laccase production implies that the model is statistically significant. There is only 0.01 % likelihood that large model F value could occur by chance. The value of p > F less than 0.05 indicates that the model terms are also significant. The R^2^ value, being a measure of the goodness of fit of the model, indicated that the 95 % of the total variation was explained by the model. The adjusted R^2^ value was 90 %. At the same time a relatively lower value of the coefficient of variation (CV = 17.47 %) indicates a better precision and reliability of the experiments carried out (Table 5).

**Table 5:** Results of the regression analysis of the central composite design (CCD)

Source	Sum of Squares	Degrees of freedom	Mean Square	F-value	*P*-value Prob > F	Significance
Model	39501352.1	9	4389039.1	20.5	<0.0001	significant
Residual	2141646.9	10	214164.7			
Lack of Fit	1749316.7	5	349863.3	4.5	0.0633	not significant
Pure Error	392330.2	5	78466.0			
Cor Total	41642999.1	19				
R^2^	0.95					

**Fig. 3:**
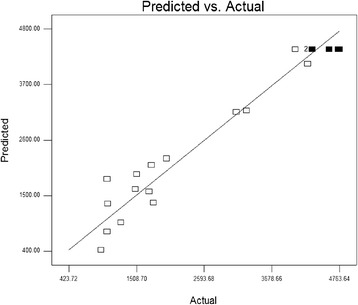
Comparison between the observed values and the predicted values of (RSM) model: zero error is shown as a *thin line*

The plot of predicted values versus experimental values in Fig. 3 also shows that all the predicted values of RSM model are located in close proximity to the experimental values. This supports the hypothesis that the model Eq. (2) is sufficient to describe the response of the experimental observations pertaining to laccase production. The Student’s t distribution and corresponding values, along with parameter estimates are given in Table 5. The ANOVA results showed that among the 22 variables, tree had significant effects (*p* < 0.05): A (casein peptone), B (Cd^2+^) and C (L/S ratio).

**Fig. 4:**
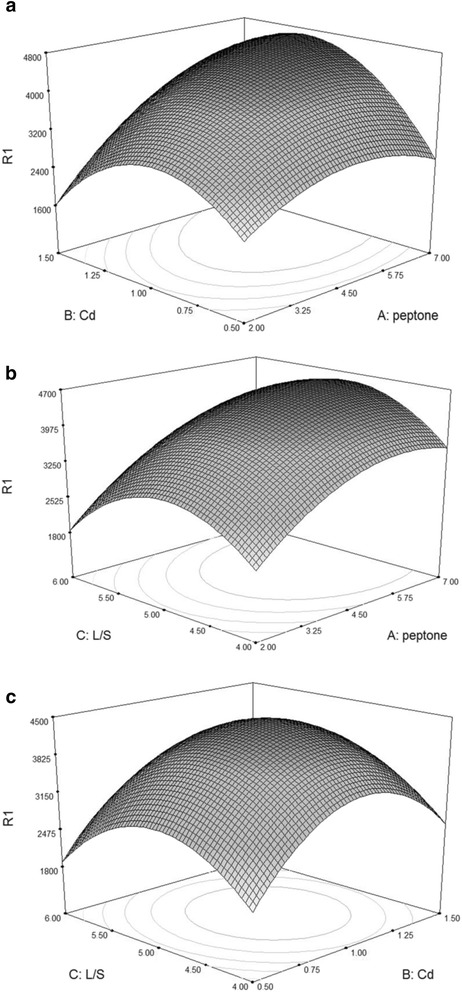
Response surface and contour plots of laccase production by *C. gallica.* under solid-state fermentation (SSF) conditions operating with sawdust as support-substrate showing the effect of two variables (other variables were kept at zero in coded unit): significant interaction between (**a**): peptone (*A*) and Cd^2+^ (*B*); (**b**): peptone (*A*) and L/S ratio (*C*); (**c**): Cd^2+^ (*B*) and L/S ratio (*C*)

**Fig. 5:**
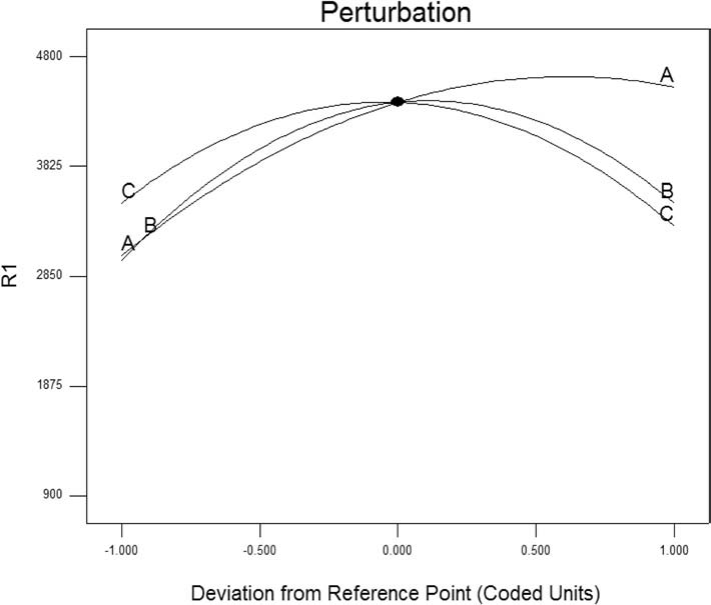
Perturbation graph showing the effect of each independent variable on laccase production while keeping other variables at their respective mid-point levels. (*A*) Casein peptone, (*B*) Cd^2+^ and (*C*) L/S ratio

The relationships between culture medium variables and response can be better understood by examining the planned series of contour plots (Fig. 4a, b and c), which were generated from the predictive model described above. Three-dimensional response surface curves were plotted to study the interaction between the two factors selected and to determine the optimum concentration of each of them for maximum laccase productivity (R1) (Fig. 4). The coded model was used to generate three-dimensional diagrams and a contour plot of calculated response surface from the interaction between casein peptone (A) and Cd^2+^ (B), casein peptone (A) and L/S ratio (C), Cd^2+^ (B) and L/S ratio (C) (Fig. 4a, b and c, respectively) as a function of the six variables being at their constant levels. The shapes of contour plots indicate the nature and extent of the interactions. The prominent interactions of significant components are shown by the elliptical nature of the contour plot in Fig. 5. The optimal values of variables are obtained when moving along the major and minor axis of the ellipse and the response at the centre point yields maximum laccase production.

The main objective of the optimization was to determine the optimum values of variables for laccase activity from the model obtained experimentally. From the response surface (Fig. 4) and perturbation plot (Fig. 5) it is obvious that casein peptone had a significant effect on laccase production compared to other variables. Figure 5 shows that the R1 response increases as the concentration of peptone from casein does. When the L/S ratio took values close to the center (4.75–5.0) and casein peptone took high concentrations (5.5 g L^−1^), laccase activity reached 4163 U L^−1^.

Cd^2+^ (C) had an acute effect on laccase activity. Figure 5c shows that the maximum laccase yield was 3992 U L^−1^ in the presence of Cd^+2^ at its central level (1 mM). However, a rapid decline in laccase productivity was observed at higher Cd^2+^ concentrations (1.25 mM). This positive effect of Cd^2+^ addition on the production of laccase was also reported by Baldrian and Gabriel [32] who found an 18.5-fold increase of laccase activity when Cd(NO_3_)_2_ (2 mM) was added to 12-day-old liquid cultures of *P. ostreatus*. In addition, Staszczak and Jarosz-Wilkołazka [33] reported that the treatment of 7-day-old mycelia with 10–200 μM Cd^2+^ resulted in considerably higher levels of laccase activity (three to five fold increase at 100 μM Cd^2+^). These results strongly support the interpretation that the proteasome-mediated proteolytic pathway plays an important role in the regulation of *C. gallica.* laccase activity in response to Cd^2+^.

The laccase activity was 4763 U L^−1^ in the optimized media with the three components at their central levels. Design Expert predicted the maximum laccase yield to be 4800 U L^−1^ in an optimized medium composed of Cd^2+^ (1 mM); casein peptone 4.5 g L^−1^, L/S ratio (5.0), MgSO_4_ (0.5 g L^−1^, KH_2_PO_4_ 0.05 g L^−1^, inoculum size 6.0 plugs and 25 mM sodium acetate pH 5.0 as buffer. Laccase yield in the optimized medium was 4800 U L^−1^, which is 3.2-fold higher than the laccase activity obtained in the initial medium (1480 U L^−1^).

The model is significant and Lack of fit is not significant thus proving the validity. The good agreement between the predicted and the experimental results verified the validity of the model, and the improvement of laccase production also indicated that RSM was a powerful tool for determining the exact optimal values of the individual factors and the maximum response value.

### Batch decolorization experiments under SSF conditions

Adsorption techniques employing low-cost adsorbents are widely used to remove certain classes of chemical pollutants from water, especially those that are practically unaffected by conventional biological wastewater treatments. Sawdust, a relatively abundant and inexpensive material is currently being investigated as an adsorbent to remove contaminants from water such as: dyes, oil, toxic salts and heavy metals [34, 35].

To test the efficiency on dye removal of the above cultures, the decolorization of RB-5 and AO51 was studied. Both RB5 and AO51 belong to the class of azo compounds which are the most widely used in textile industries. This class characterizes by one or more nitrogen-nitrogen double bonds called azo groups in their chemical structure. These molecules are chemically stable and difficult to biodegrade. Several studies reported the toxicity of RB-5 [36] and AO51 [37].

**Fig. 6:**
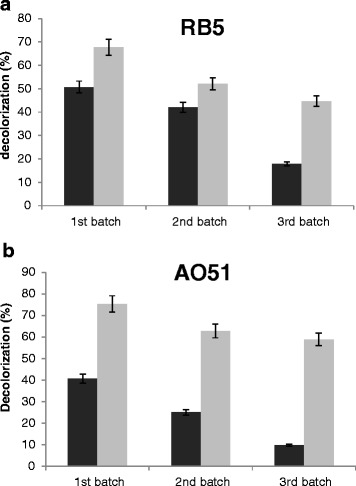
Decolorization of the azo dyes (**a**) Reactive Black 5 (RB5) and (**b**) Acid Orange 51 (AO51) by *C. gallica* cultures grown on sawdust waste under solid-state fermentation conditions in three successive batches

Figure 6a and b show the decolorization percentage obtained for each batch. The decolorization obtained in the 1st and 2nd batch was higher than that attained in the 3rd batch for RB5 and AO51. This was likely due to the rapid adsorption of the dye onto both the fungal mycelium and the sawdust waste during the first decolorization stages as it was evidenced for the change in color of the fungal mycelium and the lignocellulosic waste (40.7 and 50.8 % adsorption percentages in the 1st batch for AO51 and RB5, respectively). Once the fungal mycelium and the sawdust waste were saturated with the dye, the decolorization was only due to the action of laccase enzymes. Decolorization percentages around 67–75 % were obtained in 24 h (1st batch) for RB5 and AO51, respectively.

As is observed in Fig. 6, the differences between the controls and SSF are significant in the 2nd batch specially AO51. In the case of RB-5 the 2nd batch shows a low variation between controls and SSF cultures compared to AO51. This suggests that the decolorization of RB-5 is realized essentially by physical adsorption of the dye to the sawdust waste during the decolorization stages. However, Biological decolorization determined by subtracting the decolorization due to the physical adsorption of the dye to the support from the total decolorization was low. SSF using sawdust can be a suitable process for RB-5 decolorization. Several studies reported that the azo dye RB-5 is difficult to biodegrade and needs a mediator redox in the case of enzymatic decolorization [36, 37].

Therefore, two mechanisms were involved in dye degradation by *C. gallica* cultures: biodegradation and adsorption. The determination of the amount of dye bound to the fungal mycelium was unfeasible, since it was impossible to remove the dye from it. This might be attributed to the physical-chemical property of sawdust used as support substrate in SSF.

A similar trend toward decolorization efficiency using rice bran as a substrate for the removal of textile dyestuff from wastewater decolorization of the adsorbed textile dyes from textile wastewater was reported by Kadam et al. [38]. It was also found that decolorization degrees between 49 and 94 % could be obtained under semi-solid-state fermentation of sunflower seed shells by *Trametes pubescens* [39].

## Conclusions

Waste materials have little or no economic value and often present a disposal problem. Therefore, there is a need to valorize these low-cost by-products. So, their use as support-substrate for laccase production, add economic value and help in reducing the cost of waste disposal. The developed SSF process showed to be an effective system for sawdust waste upgrading by producing high laccase activity levels and removing azo dyes.
